# *Coccoloba uvifera* L. associated with *Scleroderma Bermudense* Coker: a pantropical ectomycorrhizal symbiosis used in restoring of degraded coastal sand dunes

**DOI:** 10.1007/s00572-024-01170-8

**Published:** 2024-10-05

**Authors:** A. M. Bâ, S. Séne, M. Manokari, M. M. Bullaín Galardis, S. N. Sylla, M. A. Selosse, M. S. Shekhawat

**Affiliations:** 1 Laboratoire de Biologie et Physiologie Végétales, Université des Antilles, Guadeloupe, France; 2 Laboratoire des Symbioses Tropicales et Méditerranéennes UMR113, UM2/CIRAD, IRD/Sup-Agro, Montpellier, France; 3 Académie Nationale des Sciences et Techniques du Sénégal, Dakar, Sénégal; 4 Laboratoire Commun de Microbiologie IRD/ISRA/UCAD, BP 1386, Dakar, Sénégal; 5 Biotechnology Unit, Kanchi Mamunivar Government Institute for Postgraduate Studies and Research, Puducherry, India; 6 Plant Biotechnology Studies Center, Faculty of Agricultural Sciences, University of Granma, Carretera Manzanillo, Bayamo, 85100 Cuba; 7 Département de Biologie végétale, UCAD, Dakar, Sénégal; 8 Institut de Systématique, UMR 7205 – CNRS, MNHN, UPMC, EPHE, Muséum national d’Histoire naturelle, Sorbonne Universités, 57 rue Cuvier, Évolution, Biodiversité, Paris, 75005 France; 9 Faculty of Biology, Department of Plant Taxonomy and Nature Conservation, University of Gdańsk, ul. Wita Stwosza 59, Gdańsk, 80-308 Poland; 10 Institut Universitaire de France, Paris, France

**Keywords:** Seagrape, *Scleroderma bermudense*, Micropropagation, Genetic diversity, Salinity tolerance, Restoration

## Abstract

*Coccoloba uvifera* L. (Polygonacaeae), named also seagrape, is an ectomycorrhizal (ECM) Caribbean beach tree, introduced pantropically for stabilizing coastal soils and producing edible fruits. This review covers the pantropical distribution and micropropagation of seagrape as well as genetic diversity, functional traits and use of ECM symbioses in response to salinity, both in its native regions and areas where it has been introduced. The ECM fungal diversity associated with seagrape was found to be relatively low in its region of origin, with *Scleroderma bermudense* Coker being the predominant fungal species. In regions of introduction, seagrape predominantly associated with *Scleroderma* species, whereas *S. bermudense* was exclusively identified in Réunion and Senegal. The introduction of *S. bermudense* is likely through spores adhering to the seed coats of seagrape, suggesting a vertical transmission of ECM colonization in seagrape by *S. bermudense*. This ECM fungus demonstrated its capacity to enhance salt tolerance in seagrape seedlings by reducing Na concentration and increasing K and Ca levels, consequently promoting higher K/Na and Ca/Na ratios in the tissues of ECM seedlings vs. non-ECM plants in nursery conditions. Moreover, the ECM symbiosis positively influenced growth, photosynthetic and transpiration rates, chlorophyll fluorescence and content, stomatal conductance, intercellular CO_2_, and water status, which improved the performance of ECM seagrape exposed to salt stress in planting conditions. The standardization of seagrape micropropagation emerges as a crucial tool for propagating homogeneous plant material in nursery and planting conditions. This review also explores the use of the ECM symbiosis between seagrape and *S. bermudense* as a strategy for restoring degraded coastal ecosystems in the Caribbean, Indian Ocean, and West African regions.

**Supplementary Information:**

The online version contains supplementary material available at 10.1007/s00572-024-01170-8.

## Introduction

Coastal ecosystems play a critical role as ecotones at the interface where land and sea interact (Farrer et al. 2022). These ecosystems span approximately 22% of the Earth’s land area and provide habitat for 38% of the global human population, including half of the world’s largest cities (Kummu et al. 2016). They can be broadly classified into four main categories: sand dunes, marshes, mangroves, and forests/shrublands. Each of these ecosystems encounters various challenges such as limited nutrient availability, elevated salinity levels, sediment movements and recurrent drought conditions (Farrer et al. 2022). Among these ecosystems, coastal sand dunes face a particular vulnerability to climate changes, alteration and destruction, largely due to the effects of urbanization driven by tourism and recreational activities (Elliott et al. 2022; Pinna et al. 2022).

There is an urgent need to restore and rehabilitate coastal sand dunes that have been lost or degraded, along with their crucial ecological functions. An effective strategy for revitalizing these habitats involves reintroducing native plant species, which make substantial contributions to the biodiversity of both fauna and flora, as well as the associated microbiota (Coban et al. 2022; Elliott et al. 2022). However, the predominant focus of research on restoration efforts has centered on plant-based approaches, with comparatively less attention dedicated to comprehending the role of microbial communities (Weidlich et al. 2020; Farrer et al. 2022). Nevertheless, it is vital to acknowledge that plants and soil microbiota coexist, forming a symbiotic consortium known as a “holobiont,” especially relevant in challenging environments (Selosse et al. 2004; Sanchez-Canizares et al. 2017; Coban et al. 2022). Indeed, soil microbiota play a pivotal role in driving the development of plant communities (Bardgett and Wardle 2010) and can thus be harnessed for the restoration of degraded soils (Coban et al. 2022).

One example of such a symbiotic relationship is the ectomycorrhizal (ECM) symbiosis, where soil fungi and fine tree roots form typical ectomycorrhizae (Van der Heijden et al. 2015). The ECM fungus provides mineral nutrients, water, and protection against biotic and abiotic stresses to the plant. In return, the plant supplies carbon to its fungal partner (Bâ et al. 2012). The ECM symbiosis is particularly important for many plant species thriving in stressful environments, facilitating primary succession on sand dunes (Koske et al. 2008), aiding in restoration after e.g. heavy metal contamination (Sousa et al. 2014), wildfires (Franco et al. 2014), or clear-cut logging (Sterkenburg et al. 2019). However, there are still knowledge gaps regarding the use of the ECM symbiosis in restoring degraded coastal sand dune ecosystems (Assad et al. 2022; Policelli et al. 2020; Weidlich et al. 2020). For instance, long-term studies are needed to assess the success of restoration through ECM plants and understand how introduced ECM fungi interact and coexist with the native microbiota community. It is also essential to determine whether these interactions directly lead to changes in plant productivity. These efforts are crucial for promoting the use of ECM fungi in coastal zones (Policelli et al. 2020; Zhu et al. 2022).

The degradation of coastal forest ecosystems in the Caribbean, Indian Ocean, and West Africa, driven by increasing salinity and harmful human activities, poses a significant threat to biodiversity and hinders forest regeneration, thereby compromising vital ecosystem services. (ANCORIM 2017). The current restoration approach in those regions, entails the planting of indigenous or exotic plants in degraded coastal forest ecosystems. Among these, *Coccoloba uvifera* L., commonly known as seagrape, emerges as a promising candidate for ecological restoration purposes. This Caribbean tree species demonstrates remarkable tolerance to salt, drought and wind, rendering it well-suited for coastal ecosystems. Furthermore, seagrape plays a pivotal role in stabilizing beaches and dunes and is frequently employed as a windbreak and as an ornamental plant (Parrotta 2000; Séne et al. 2015; Séne et al. 2018; Bullain Galardis et al. 2023; Manokari et al. 2023). Moreover, seagrape provides edible fruits for both humans and animals such as birds, and serves a crucial ecological function in the protection of nesting sea turtles on beaches by mitigating the adverse effects of artificial lighting emanating from the shoreline (Tuxbury and Salmon 2005).

ECM fungal diversity associated with the Polygonaceae *C. uvifera* was found to be relatively low in both its native and introduced coastal regions (Bâ et al. 2014; Séne et al. 2015, 2018; Põlme et al. 2017; Bullain Galardis et al. 2024) compared to the ECM fungal diversity observed in African tropical forests (Diédhiou et al. 2010; Bâ et al. 2012; Ebenye et al. 2016) and in the Caribbean islands (Miller et al. 2000). It is also well known that one of the dominant ECM fungi on seagrape, *Scleroderma bermudense* Coker, is tolerant to salt stress in pure culture (Bâ et al. 2014) and can help seedling seagrape to mitigate salt stress in nursery and field conditions (Bandou et al. 2006; Bullain Galardis et al. 2022; Bullain Galardis et al. 2023). Here, we review the pantropical distribution and micropropagation of seagrape as well as genetic diversity, functional traits and use of ECM symbioses of seagrape in response to salinized sandy soil, both in their regions of origin and of introduction. We focus on the growth and physiological responses to salt stress of the ECM symbiosis between seagrape and *S. bermudense* under nursery and field conditions. The ultimate goal of this review is to discuss the potential role of the ECM symbiosis between seagrape and *S. bermudense* in restoring of degraded coastal sand dune ecosystems in the tropical Caribbean, Indian Ocean and African regions.

## A pantropical distribution of seagrape

The genus *Coccoloba* belongs to the family of Polygonaceae. This plant family, containing around 1,200 species distributed over 48 genera (Abdel Hakim et al. 2019), has two ECM genera, *Coccoloba* and *Polygonum*, rich of 120 to 130 species, respectively (Howard 1988; Abdel Hakim et al. 2019). *Coccoloba* includes several species that predominate in South America, the largest number of species being in Brazil. The genus *Coccoloba* is represented at least by twenty-three species including seagrape in Brasilian Amazonia (Melo 2004; Melo et al. 2019). Of the *Coccoloba* species found in the Lesser Antilles, 10 plant species have been identified, including 6 species from Guadeloupe and Martinique (Table S1). In the island of Cuba, 34 species of *Coccoloba* are known, including seagrape, and 25 of them are endemic, placing Cuba as an important center of diversity of this genus in the Antilles (Castañeda 2014).

**Fig. 1:**
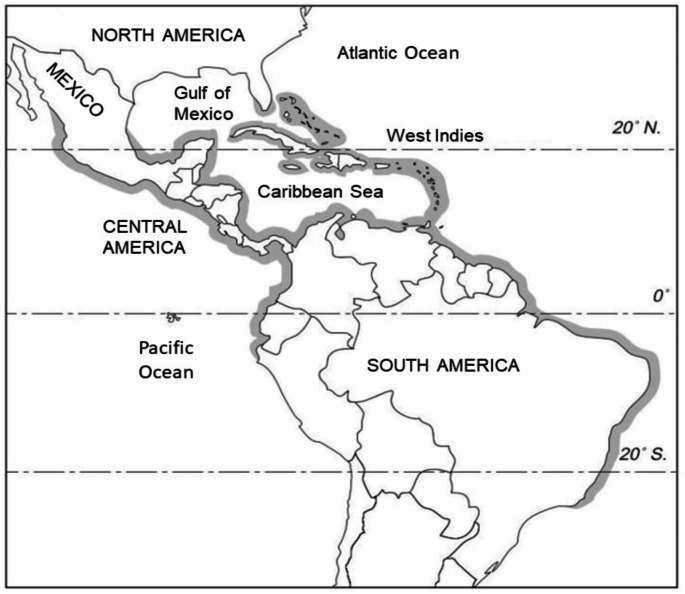
Natural distribution of seagrape indicated by the shaded areas along the coasts of the Americas and the Caribbean islands (Parrotta 2000)

Seagrape is native to the coasts of southern Florida, Bermuda, the Bahamas, the West Indies, northern and eastern South America to Northern Brazil, Mexico, and Central America and the Pacific coast of South America to Peru (Fig. 1) (Record and Hess 1943; National Academy of Sciences 1983; Melo 2004). It was introduced to the Philippines and Zanzibar during the 1940s and more recently in coastal areas in the Indian Ocean (e.g. Reunion), West Africa (e.g. Senegal), and Asia (e.g. Japan, India, Malaysia) where it is planted as an ornamental and in coastal windbreaks (National Academy of Sciences 1983; Sène et al. 2018). The native range of seagrape includes the tropical very dry, dry, and subtropical moist forests life zones (Holdridge 1967). Within its distribution, the average annual precipitation varies between approximately 500 and 1600 mm without a dry season or with a dry season lasting up to 8 months (Von Carlowitz 1986). Throughout its distribution, the average annual temperatures during the warmer months average around 28 °C. During the colder months, average temperatures range from 18 ° C in the north to 26 ° C in the south (Hoffman 1975; Steinhauser 1979). In South Florida, seagrape is subject to very occasional frosts too (National Academy of Sciences 1983).

Seagrape is one of the first species to colonize the rocky and sandy coasts (Parrotta 2000). It is tolerant of salt and grows well on almost pure sands and rocky substrates. It can survive on calcareous substrates, including limestone, and on dry or very wet substrates derived from igneous rocks, provided these sites have good drainage. It grows best in well-drained loamy sands with pH values higher than 7.5. It is usually limited to coastal areas and rarely found in inland forests. In Cuba and Jamaica, where the seagrape shows its best growth, it is found in humid forests up to an elevation of 150 m (Record and Hess 1943; National Academy of Sciences 1983; Von Carlowitz 1986; Graham and Miller 2005).

## Main uses of seagrape

### Interests and phyto-constituents

Seagrape has been traditionally used as medicine from ancient times (Panga 2014; Abdel Hakim et al. 2019). The fruits are juicy eaten raw and used to make various edible and toiletry products (Facciola 1998). This plant is a good source of different types of phytochemicals like α-amyrin, β-sitosterol, tannins, rhein, emodin, royleanone, anthraquinones, volatile oils, etc. (Silveira et al. 2008; Rodriguez-Garcia et al. 2019). The phytoconstituents of fruits have free radicals scavenging and antioxidant activities (Bailey et al. 2011; Campos et al. 2015). Jamaica Kino or American Kino is a juice from this plant used to treat diarrhoea and dysentery (Alexander and Parker 1994). Seagrape extracts showed antidiabetic, antibacterial, antiviral, and antioxidant properties (Silveira et al. 2008; Rodriguez-Garcia et al. 2019). The leaf and wood decoctions are used to treat skin diseases, anemia, tumors and asthma, and the root extracts work against skin rashes, diarrhea, hemorrhages, etc. (Usvat 2015). The fruits contain vitamins and are used to make fermented products such as drinks and wine, and other edibles like jams, jellies, and soups (Campos et al. 2015). In the West Indies and Jamaica, the tannins obtained from the stem and root bark of seagrape is used in tanning and dyeing factories (Barwick 2004).

### In vitro culture studies

**Fig. 2:**
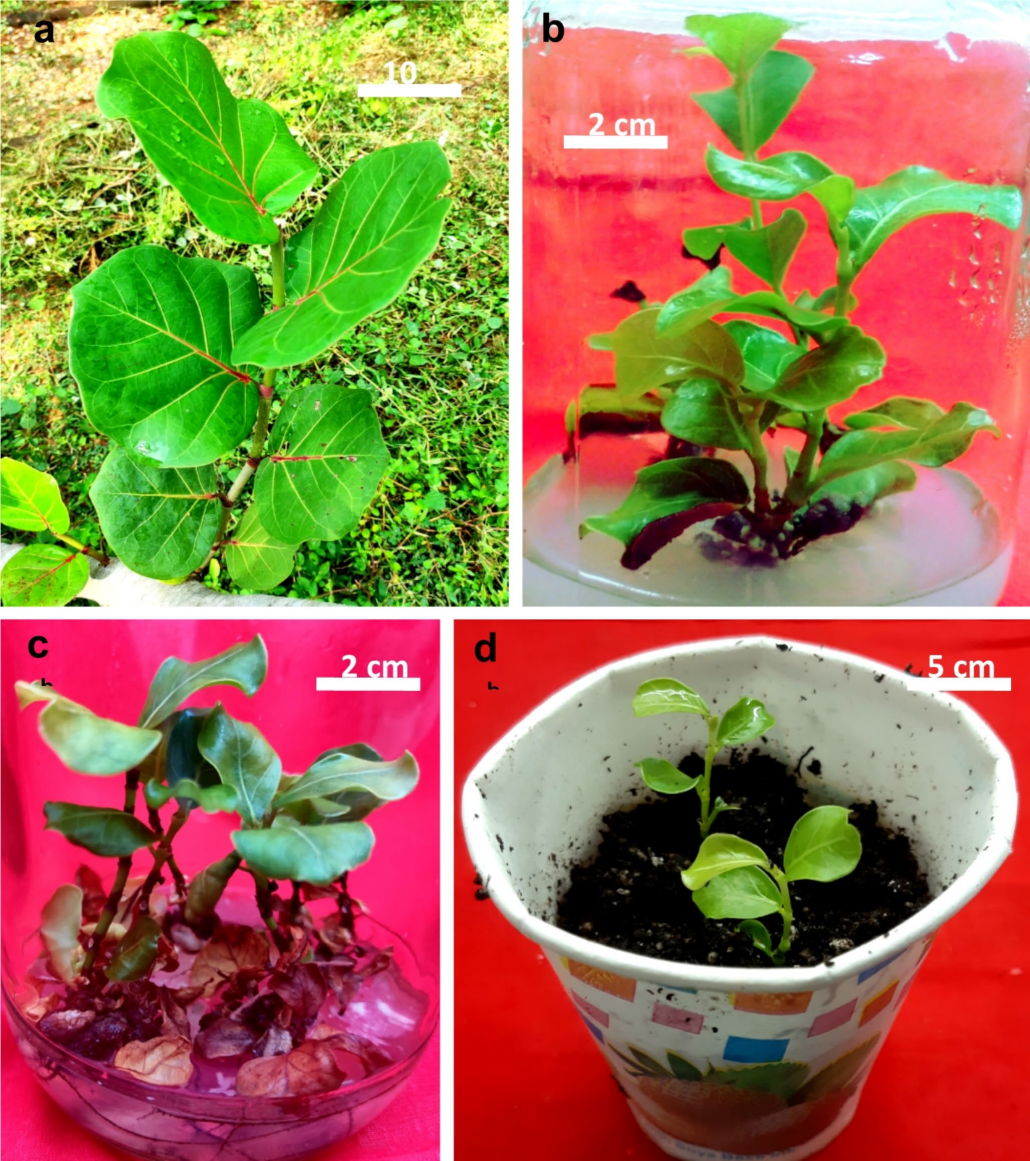
Different stages in seagrape micropropagation. **a**, fresh branch used to prepare explants, **b**, shoot multiplication. **c**, rooting of shoots. **d**, hardening of plantlets in the greenhouse (Manokari et al. 2020)

In vitro regeneration from vegetative tissues has not been carried out in this species. Manokari et al. (2020) standardized a micropropagation system using axillary buds as explants collected from the mature seagrapes, using Murashige and Skoog’s (MS) medium supplemented with 3.0 mg L^− 1^ 6-benzylaminopurine (BAP) (Fig. 2). They observed that MS medium fortified with BAP + α-Naphthalene acetic acid (NAA) with additives (ascorbic acid + adenine sulphate + citric acid + arginine) allowed better shoot multiplication. They reported callus formation with a higher amount of auxin in the medium and obtained in vitro shoot formation on diluted ½ MS medium containing NAA. *Ex vitro* rooting was successful in greenhouse using 400 mg L^− 1^ NAA in a soil mixture (Manokari et al. 2021).

Manokari et al. (2021) also tested the impact of the polyphenol compound phloroglucinol (PG) on shoot multiplication and development of morpho-biochemical features in seagrape while tissue culturing it. Phloroglucinol at 0.5-2.0 mM in the MS medium improved morphometric and biochemical parameters of the in vitro multiplied shoots. It also increased the length of shoots, foliar biomass, photosynthetic pigments, carbohydrate and protein contents. The authors reported that PG treated shoots showed better rooting and a higher percentage of survival of plantlets after transplantation in the greenhouse and field conditions (Manokari et al. 2021). Another attempt of in vitro propagation of this plant by Manokari et al. (2023) used Polyethylene-glycol (PEG) to induce drought stress in the medium to improve the quality of in vitro shoots and leaf structural and histochemical traits. They reported that 200 mg L^− 1^ PEG induced healthy shoots of seagrape with leaves having well-differentiated cuticle, ground (sclerenchyma, collenchyma and parenchyma) and vascular tissues as compared to the untreated cultures. They found that PEG is a promising stress stimulator to improve shoot and leaf morpho-anatomy and histochemical accumulation for survival under harsh greenhouse and natural environments (Manokari et al. 2023).

## ECM fungal diversity of seagrape in regions of origin

### A species-poor ECM fungal community

Many trees form ECM symbioses with diverse Basidiomycota and Ascomycota, play a key role in many tropical forests (Bâ et al. 2012; Corrales et al. 2018), affecting tree growth and nutrient absorption as well as protection against pathogens (Smith and Read 2008; van der Heijden et al. 2015). Large areas of tropical and subtropical forests are dominated by ECM trees (Bâ et al. 2012; Corrales et al. 2018), suggesting a key role of these symbioses in their ecological functioning. ECM surveys from tropical forests show that Africa has the highest number of confirmed ECM plants, followed by the Neotropics, including Central and South America and the Caribbean (Corrales et al. 2018). ECM host species in the neotropical lowlands are from predominantly tropical lineages in the Fabaceae, Cistaceae, Dipterocarpaceae, Polygonaceae, and Nyctaginaceae families (Corrales et al. 2018).

In the Lesser Antilles (e.g. Guadeloupe and Martinique) and the Greater Antilles (e.g. Cuba), the ECM fungal diversity associated with the Polygonaceae seagrape was quite poor (Bâ et al. 2014; Séne et al. 2015, 2018; Bullain Galardis et al. 2024) as compared to ECM fungal diversity in tropical forests of Africa (Diédhiou et al. 2010; Bâ et al. 2012; Ebenye et al. 2016; Rivière et al. 2017), Central and South America (Henkel et al. 2005; Peay et al. 2017; Corrales et al. 2018). This low diversity does not result from insufficient sampling, because species accumulation curves of sporocarps and ECM fungal taxa reached an asymptote at all sites in Guadeloupe (Séne et al. 2015). Our studies have once confirmed that belowground ECM fungal diversity from ectomycorrhizae is dissimilar from that of aboveground sporocarps (Séne et al. 2015; Ebenye et al. 2016; Bullain Galardis et al. 2024), as is commonly described (Richard et al. 2005), thus underlying the importance of molecular assessment of ECM fungal diversity. Other Caribbean and South American studies confirmed this feature which can be generalized to the whole distribution area of seagrape (Bâ et al. 2014; Põlme et al. 2017). However, the low diversity of ECM fungi from sporocarps and ectomycorrhizae observed in the Antilles may be the result of the adverse environmental conditions in which seagrapes grow, i.e.sandy, calcareous and rocky soils with a low percentage of organic matter and high levels of salinity (Parrotta 2000; Séne et al. 2015). Another possible cause may be the fact that seagrape is the only ECM host in coastal vegetation (Séne et al. 2015; Bullain Galardis et al. 2024). Ishida et al. (2007), hypothesize that ECM fungal diversity is driven by the number of host tree species in a particular ecosystem. Indeed, seagrape is the only ECM host of the investigated beach sites. Finally, the ECM association is a rather recent evolution in two clades of the Polygonaceae, a predominantly non-mycorrhizal family (Tedersoo and Brundrett 2017) and the genus *Coccoloba* diverged from non-ECM clades around 52 Ma ago and radiated 24 Ma ago (Schuster et al. 2013): this may explain that species of this genus evolved a limited compatibility to only few ECM fungi (Table S2).

Overall, *S. bermudense* was the alone ECM fungus that was found both as sporocarp and on ectomycorrhizae, whatever the sites. In all, seagrape is associated with ECM fungal species belonging to the genera *Amanita*, *Inocybe*, *Cantharellus*, *Melanogaster*, *Cenococcum*, *Lactarius*, *Russula*, *Thelephora*/*Tomentella*, *Tuber*, *Xerocomus* and *Scleroderma* (Kreisel 1971; Pegler 1983; Miller et al. 2000; Guzmán et al. 2004; Álvarez 2012; Bâ et al. 2014; Séne et al. 2015, 2018; Bullain Galardis et al. 2024). Furthermore, phylogenetic analysis demonstrate that the different species fall in 7 ECM fungal lineages proposed by Tedersoo et al. (2010), including 6 from basidiomycetes (/russula-lactarius-lactifluus, /amanita, /cantharellus, /thelephora-tomentella, /pisolithus-sclerodema, and /inocybe) and 1 from an ascomycete lineage (/tuber-helvella) (Séne et al. 2015; Bullain Galardis et al. 2024). Based on phylogenetic analysis and % of similarity (99–100%), only three common ECM fungi, *S. bermudense*, *Inocybe* sp. and *Thelephora* sp. from Cuba matching with *S. bermudense*, *Inocybe xerophytica* and *Thelephora* sp. from Guadeloupe, respectively. It is also interesting to note that two *Tuber* spp. were identified molecularly from ectomycorrhizae collected in Cuba. However, *S. bermudense* was the most abundantly fruiting fungal species found in close proximity to seagrape and represented the predominant ECM fungus on roots of the seagrape in the littoral forests of the Greater Antilles (Cuba) and the Lesser Antilles (Martinique and Guadeloupe) (Bâ et al. 2014; Séne et al. 2015; Bullain Galardis et al. 2024).

### Shared ECM fungi by seagrape seedlings and mature trees

Seagrape forms major stands growing in littoral forests with abundant seedling recruitment in the mother tree’s crown (Séne et al. 2015; Séne et al. 2018; Bullain Galardis et al. 2024). The high regeneration of seagrape seedlings beneath mature trees can be attributed to the fact that the mature trees serve as a source of ECM fungal inoculum for the seedlings through common mycorrhizal networks (CMNs). Indeed, seagrape seedlings and mature trees had very similar communities of ECM fungi based on molecular analysis of ectomycorrhizae and could share potential CMNs (Séne et al. 2015; Bullain Galardis et al. 2024). Among the shared ECM taxa, *S. bermudense* followed by Thelephoraceae species, were the most frequent on root tips of seagrape seedlings and mature trees: they may form a CMN between these cohorts in the Lesser Antilles (Séne et al. 2015) and Greater Antilles (Bullain Galardis et al. 2024), resulting in a nursery effect. It is also noted that two ECM fungal taxa belonging to the genus *Tuber* were found for the first time to be shared by seagrape seedlings and mature trees in the Greater Antilles (Bullain Galardis et al. 2024). Previous studies in tropical rain forests also found low diversity of ECM ascomycetes (Diédhiou et al. 2010; Henry et al. 2015; Ebenye et al. 2016). ECM basidiomycetes such as *S. bermudense* were the most representative fungal taxa on roots of mature trees and seedlings whatever the studied site. They may be important for the nursery effect. Similarity of ECM fungal taxa composition between mature trees and seedlings has often been reported in temperate (Aučina et al. 2011) and tropical forests (Diédhiou et al. 2010; Séne et al. 2015; Ebenye et al. 2016). For instance, mature trees and seedlings of dominant seagrape coastal forests shared three ECM fungal taxa representing 80% of the ECM colonization with a predominance of *S. bermudense* (Séne et al. 2015). In a mixed tropical rain forest in Guinea, ECM fungi shared by mature trees and seedlings represented 79% of the ECM colonization (Diédhiou et al. 2010). The CMNs and their impact on the nutrition, growth, and fitness of regenerating seedlings should be further investigated experimentally in seagrape coastal forests.

## Diversity of ECM fungi on seagrape in regions of introduction

### Intraspecific diversity and host preference of *Scleroderma bermudense* on seagrape

Seagrape is a pantropical tree distributed around the world for ornamental and windbreak purposes in countries such as India, Malaysia, Japan, Reunion, and Senegal (Manokari et al. 2020; Sène et al. 2018). In the latter two, it is extensively planted to diversify plantations of exotic trees, including *Eucalyptus camaldulensis* and *Casuarina equisetifolia* (Séne et al. 2018). In Reunion and Okinawa, seagrape has been introduced as an ornamental plant and to reinforce rocky strips along the coastline. In Senegal, seagrape is used as an ornamental plant and to fix sand dunes to combat silting of roads along the coast.

Sène et al. (2018) investigated (i) which ECM fungi were co-introduced with seagrape, (ii) whether *S. bermudense* populations from Reunion and Senegal are differentiated from Caribbean populations, and (iii) whether the introduced *S. bermudense* populations are compatible with the pre-existing host trees in the areas of introduction. *S. bermudense* should possibly be considered as a Caribbean endemic species and has exclusively been documented beneath seagrape. Genotyping with microsatellite markers was used to verify whether *S. bermudense* individuals recruited in the introduction regions were co-introduced, or were of local origin (Séne et al. 2018). The results showed low genetic diversity within *S. bermudense* populations in the introduced areas (Senegal and Reunion), with an absence of private alleles, compared to the genetic diversity observed in its native area (Guzmán et al. 2004; Põlme et al. 2017; Séne et al. 2018). One cannot exclude that *S. bermudense* is a previously unnoticed pantropical species, whose visibility became more prominent following the introduction of seagrape. This could potentially account for the absence of a founder effect in Senegal and Reunion. A population genetic analysis of *S. bermudense* revealed no evidence of a demographic bottleneck linked to a potential founder effect. However, fungal populations of *S. bermudense* from regions where seagrape has been introduced show low genetic differentiation from those in the Caribbean, despite being separated by thousands of kilometers. This aligns with a relatively recent introduction of seagrape (Séne et al. 2018).

In Reunion and Senegal where *E. camaldulensis* and *C. equisetifolia* adults co-occur with seagrape, conspecific seedlings were colonized by several ECM fungi including *Scleroderma bovista* and *Pisolithus* sp. but not *S. bermudense*, whereas coexisting seagrape seedlings were exclusively colonized by *S. bermudense*. Conversely, under seagrape adults, *E. camaldulensis* and *C. equisetifolia* seedlings were not mycorrhizal, whereas seagrape seedlings were again colonized by *S. bermudense*. This suggests a reciprocal preference of seagrape and *S. bermudense* in all studied forest sites (Séne et al. 2018). Changes of host plant are frequent in ECM fungi when they are introduced into new habitats, particularly in mixed plantations where the roots of different trees intermingle (Jairus et al. 2011). This suggests a rather strong host preference of *S. bermudense* for seagrape in Senegal and Reunion, also in areas of introduction. In fact, *S. bermudense* is a fungal symbiont of seagrape, whose distribution seems to follow seagrape in areas of origin and introduction (Guzmán et al. 2004; Bandou et al. 2006). Seagrape may therefore need *S. bermudense* to better establish itself in a new habitat, since the success of tree plantations is strongly linked to the presence of ECM fungi: for example, *Rhizopogon* and *Suillus* spp. are the main pine-specific fungal ECM symbionts and their absence can lead to the failure of pine plantations in new habitats. The low diversity of ECM fungal associates of *C. uvifera* in its areas of introduction in Africa (Senegal) and Asia (Japan and Malaysia) would be due to the absence of compatible local ECM symbionts. This low diversity could also be due to the specificity of the habitat’s introduction of *C. uvifera* which would be less adapted to the ECM fungal symbionts of origin of this plant, with the exception of *S. bermudense*.

### A scenario of co-introduction of seagrape and *S. bermudense*

**Fig. 3:**
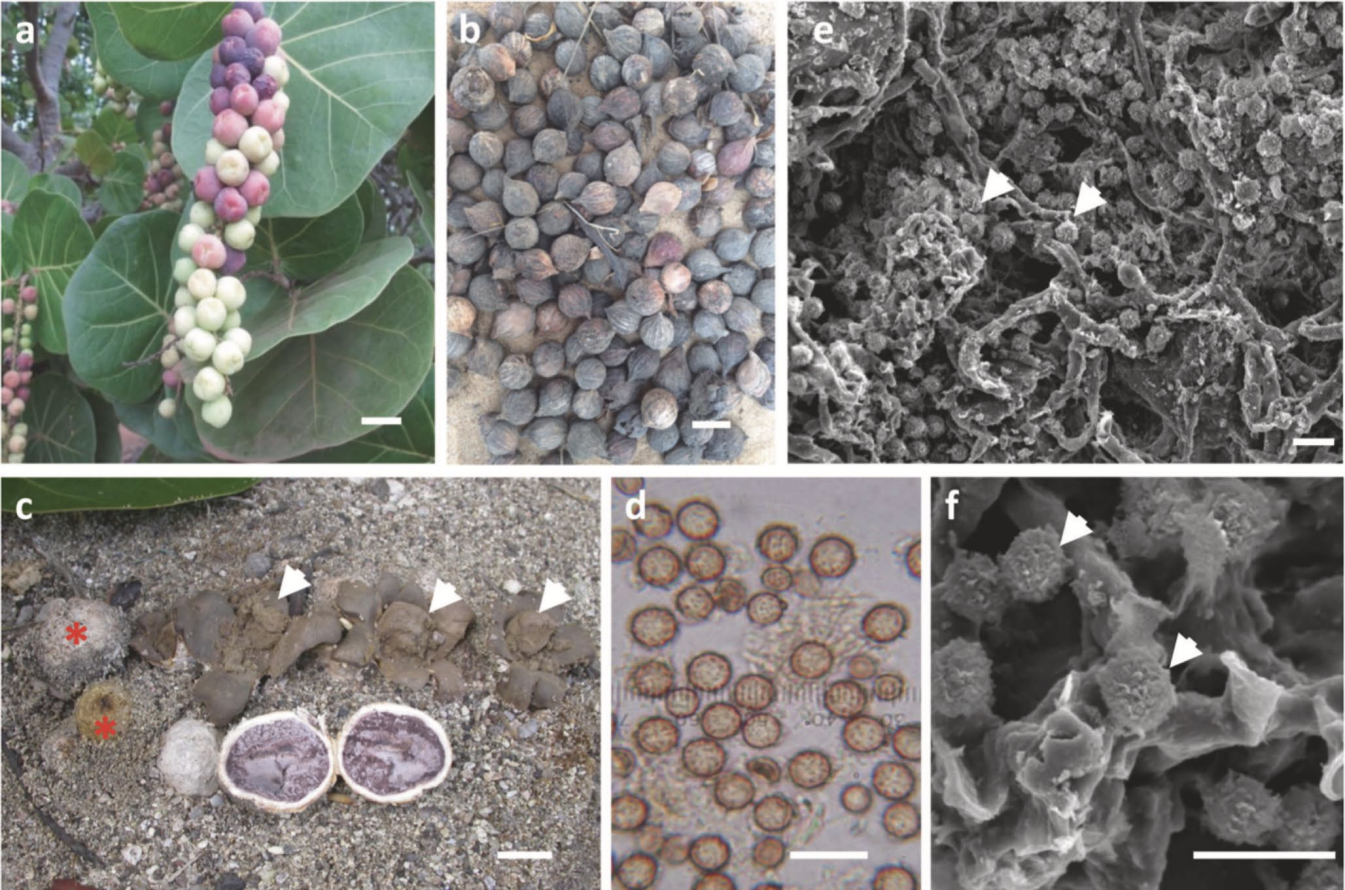
Seagrape fruits and *Scleroderma bermudense* spores from Bois Jolan (Guadeloupe). (**a**) Fresh and (**b**) dry seagrape fruit; (**c**) *S. bermudense* sporocarps, either semi-hypogeous and immature (red asterisks), or old and releasing spores (arrowheads), with a section of a young sporocarp displaying the violet immature spore mass; (**d**) *S. bermudense* spores in light microscopy; (**e**), (**f**) SEM of pericarp surface from a fruit covered by *Scleroderma* spores (arrowheads). Bars are 1 cm in (a-c); 10 μm in (d-f) (Séne et al. 2018)

Seagrape exhibits a specific association with *S. bermudense* in Senegal, where the latter predominantly colonizes the plant’s roots (Séne et al. 2018). In Reunion, *S. bermudense* also dominates the root systems of seagrape (Séne et al. 2018). We hypothesized that *S. bermudense* was introduced from the Caribbean, most likely through spores adhering to the seed coats of seagrape. Indeed, a vertical transmission of *S. bermudense* was demonstrated as follow: mature fruits of seagrape fall down and while drying on beach soil, they aggregate spores from the spore bank accumulated by semi-hypogeous *S. bermudense* sporocarps, so that finally spores of *S. bermudense* are incrusted in seeds (Fig. 3). When seeds were transported to introduction sites, spores sticking on seed coats germinated and colonized seagrape roots in nurseries and other planting sites (Séne et al. 2018). Additional experiments confirmed the potential of this spore bank as an inoculum (Séne et al. 2018). The observed co-adaptation between seagrape and *S. bermudense*, spanning different regions of introduction, strongly indicates a cooperative relationship between these two symbionts. This cooperative bond is likely fortified through vertical transmission, favoring coevolution. This mechanism not only contributes to host preference but also plays a vital role in strengthening the mutualistic association between the two partners. It is noteworthy that vertically transmitted symbioses typically exhibit greater stability and altruism (Provorov and Vorobyov 2010). As a consequence, seedlings tend to form associations with spores of *S. bermudense* that are genetically similar to the mycelia colonizing the mother trees, preserving genetic combinations across successive generations. This process is believed to have facilitated the development of mutualistic traits, including specificity, growth enhancement, and protection against salt (Séne et al. 2018).

The exotic fungal species *S. bermudense* persists with introduced seagrape and does not spread to native host plants (Séne et al. 2018). Despite the high frequency of ECM fungal introductions, little data exist on the potential effects of these introductions on the diversity of native plant communities (Desprez-Loustau et al. 2007). However, Richardson et al. showed that introduced ECM fungi can facilitate the invasion of introduced plant species. This is the case of invasion of exotic *Pinus* species in the fynbos of southern Africa which was facilitated by ECM fungi. Such introduced ECM fungi can harm native plant species or alter nutrient cycles in soils (Vellinga et al. 2009).

## Tolerance of the ECM symbiosis between seagrape and *S. bermudense* to salt stress

**Fig. 4:**
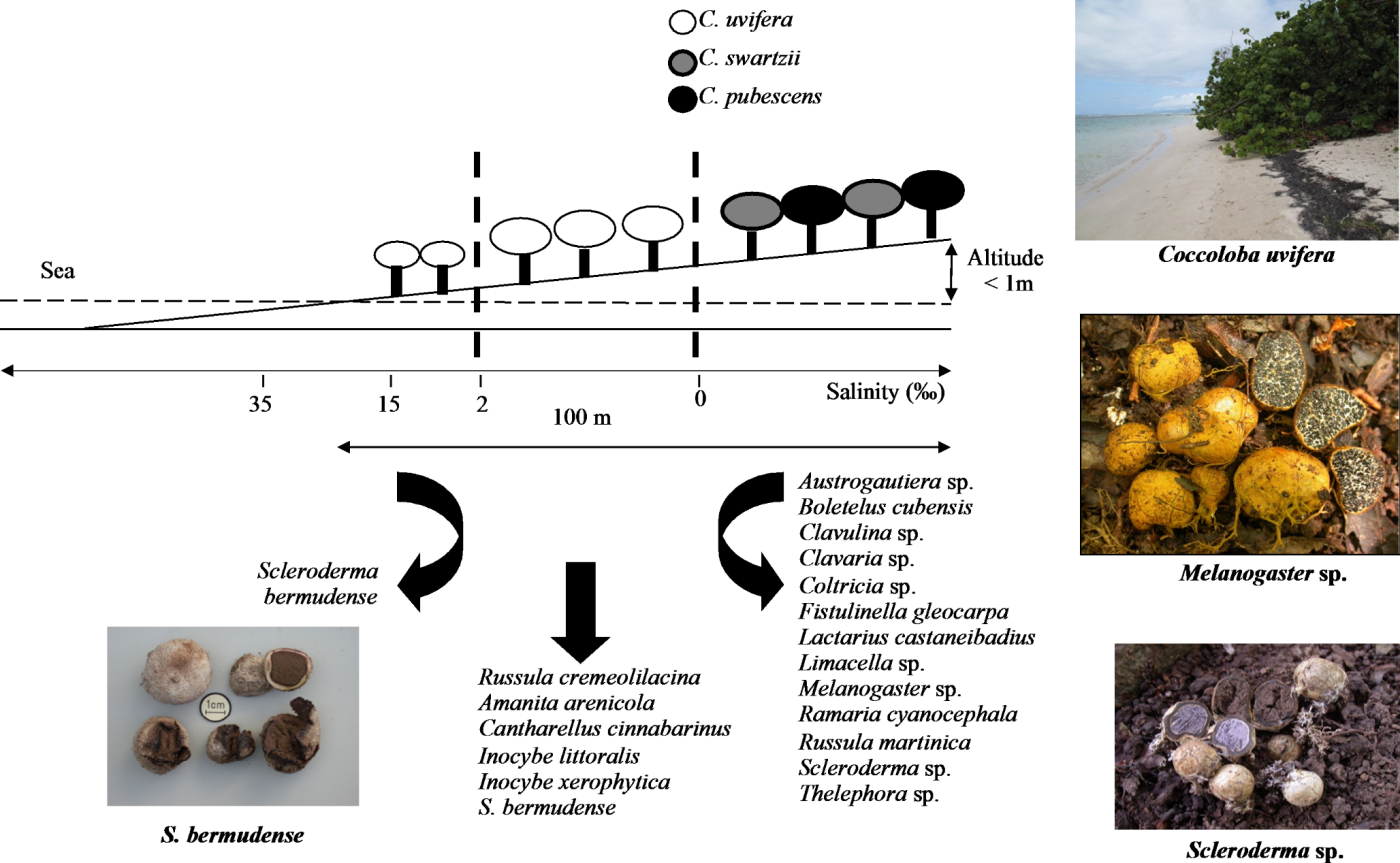
Distribution of ECM fungi associated with *Coccoloba uvifera*, *C. swartzii* and *C. pubescens* along a salinity gradient in Martinique and Guadeloupe (Bâ et al. 2014)

In the Lesser Antilles (Guadeloupe and Martinique), of the six species of *Coccoloba* identified (Table S1), three were more abundant: *C. uvifera*, *C. pubescens* and *C. swartzii* (Fig. 4). *C. pubescens* also named ‘grandleaf seagrape’ (leaf diameter 2.5–45 cm) is distributed in the xero-mesophytic forests in Guadeloupe and Martinique, whereas *C. swartzii* is encountered in the degraded xerophytic forests in the both islands. *C. pubescens* and *C. swartzii* co-occur often together in Martinique. Given the natural distribution of the three species of *Coccoloba* along a salinity gradient (Fig. 4), *C. uvifera* seems to be better adapted to salinity than the others. Mature trees of *C. uvifera* with abundant seedling recruitment were well developed within areas far from the sea where salt levels were low (0–2‰), whereas mature trees in salty sand near the sea (2–15‰) were shunted and seedlings were almost absent (Fig. 4). Diversity of ECM fungi increased when the salinity was lower: six species (sporocarp observations) were identified at low salinity levels (0–2‰) and only *S. bermudense* fructified at the high salinity level (2–15‰). This suggests that the latter ECM fungus is best adapted to the salinity stress (Fig. 4). Furthermore, naturally regenerating seedlings under the canopy of mature trees were high at low salinity levels, whereas very little occurred under the mature trees growing near the sea (Bâ et al. 2014). Salinity is considered as one of the most significant environmental factors limiting plant germination, growth and productivity (Flowers and Colmer 2008). Salinity reduces availability of water and increases toxic ion concentrations in soils. However, the way in which salinity exerts its influence on these vital processes, through an osmotic effect or a specific ion toxicity, is still not resolved.

### Tolerance of *S. bermudense* to salt stress in pure culture

Among the sporocarps collected from under the three *Coccoloba* species in French Lesser Antilles, only three of them (*S. bermudense*, *Scleroderma* sp. and *Melanogaster* sp.) were successfully isolated and cultivated in a MMN medium (Marx 1969) in pure culture (Bâ et al. 2014). Two fungal species (*Scleroderma* sp. and *Melanogaster* sp.) were from a non-salt-affected soil, and one (*S. bermudense*) was from salt-affected soil along a salinity gradient (Fig. 4; Figure S5). *S. bermudense* performed better in pure culture than *Melanogaster* sp. and *Scleroderma* sp. whatever the levels of NaCl (Figure S5). Furthermore, *S. bermudense* accumulated less Na^+^ and more proline than the other two ECM fungi, suggesting a Na^+^ exclusion mechanism and an osmoregulation mechanism under low water potential, respectively.This suggests that *S. bermudense* creates a stronger barrier against salinity stress than the other fungi. Similarly, Chen et al. (2001) reported that certain fungal isolates of *Pisolithus* exhibited distinct peaks in biomass yield within the concentration range of 25–100 mM NaCl. ECM fungi demonstrate a capacity to endure elevated osmotic potential and toxic metal ions by employing a strategy that involves the compartmentalization of toxic ions and the production of polyols, which function as non-toxic osmoregulators (Bois et al. 2006a, b; Chen et al. 2001).

### Tolerance of seagrape associated with *S. bermudense* to salt stress in nursery conditions

Globally, salinity affects a staggering 800 million hectares of soil (Munns and Tester 2008), encompassing more than 6% of the Earth’s land and roughly 20% of cultivated areas. Disturbingly, the extent of land affected by salinity could increase to 50% of the total cultivated area by 2050 (Courel 2019). Soil salinization, resulting from both natural and human-induced factors, entails the excessive accumulation of salts in the soil. This challenge predominantly plagues arid, semi-arid regions, and coastal areas due to improper irrigation practices employing low-quality groundwater, salt resurgence *via* capillarity, and rising sea levels attributed to climate change (Gamalero et al. 2009; Gopalakrishnan and Kumar 2021). The presence of elevated salt concentrations in soils has detrimental consequences for biodiversity, agricultural productivity, and the pursuit of sustainable development (Tester and Davenport 2003).

Several strategies have been established to overcome salt-stress problems such as a selection of salt-tolerant plants, a desalination of soil by leaching excessive salts and using mycorrhizal interactions (Chen et al. 2017). Indeed, ECM symbiosis is a key factor for improving tolerance of some woody plants to salt stress through three mechanisms: (i) exclusion and compartmentation of Na in an extracellular hyphal network, (ii) improving K uptake in host plants and (iii) improving the water status of plants through the activation of both fungal and plant aquaporins (Bandou et al. 2006; Guerrero et al. 2019; Zwiazek et al. 2019). This leads to a higher K/Na ratio in ECM than in non-ECM plants under salt stress (Bandou et al. 2006; Guerrero et al. 2019).

Total biomass tended to diminish similarly in ECM plants *versus* non-ECM plants under saline conditions regardless of seagrape provenances inoculated with spores (Table S3). As a consequence of this, ECM dependency apparently increased with increasing NaCl levels (Table S3). This suggests that ECM plants mitigated salt stress in both seagrape provenances and indicates a high symbiotic efficiency of *S. bermudense* once it was established. A similar effectiveness of the ECM symbiosis was also reported in other tree species, including *Pinus* spp. For example, Zwiazek et al. (2019) found that *Pinus concorta* was dependent on ECM fungi that could be helpful in alleviating effects of NaCl in urban soils. Bandou et al. (2006) had also shown an increase of ECM dependency with an increasing salt level within in one seagrape provenance from Guadeloupe.

Although salinity reduced stomatal conductance (gs), photosynthetic and transpiration rates, and sub-stomatal CO_2_ in both non-ECM and ECM plants, these parameters remained higher in ECM than in non-ECM plants under saline conditions regardless of seagrape provenances (Table S4). The result suggests that ECM fungi can elevate the photosynthetic ability of seagrape seedlings by improving stomatal conductance and gas exchange capacity under salt stress (Ashraf and Harris 2013). It is consistent with work of Shi-chu et al. (2019) who showed that increasing salt concentration led to a significant decrease of photosynthetic rate *via* a decrease in the gs, which was less important in arbuscular mycorrhizal than in non-mycorrhizal alfalfa. The decrease of gs due to the stomatal closure is often related to the water status of plants (Mohamed et al. 2020). Under water deficit conditions due to salt stress, seagrape reduced transpiration rate more in non-ECM than in ECM plants by closing stomata to reduce water loss. Furthermore, a higher gs in the ECM vs. non-ECM seagrape plants under salt stress may be related to the increased CO_2_ diffusion through the stomata and water absorption (Table S4).

Measurements of relative water content (RWC), foliar potential water (Ψwf) and xylem potential water (Ψwx) are considered informative on water status and transpiration rate in plants under salt stress (Larcher 2003; Chen et al. 2017). Despite their higher evaporative leaf surface, ECM seagrape had higher RWC, Ψwf and Ψwx than non-ECM plants regardless of salinity and seagrape provenances (Table S5), suggesting that the extensive hyphal extension of ectomycorrhiza allows higher water absorption and hydraulic conductivity even when water potential is low (Bandou et al. 2006; Augé et al. 2008; Lehto and Zwiazek 2011).

ECM plants kept a higher K/Na ratio in leaves, shoots and roots and confirmed that K competed for the absorption site of Na on the cell membrane (Table S6). Therefore, ECM symbiosis can facilitate K whereas preventing Na absorption and translocation in shoot and leaves of seagrape seedlings to maintain a high cytosolic K/Na ratio which is a key feature of plant salt tolerance. Our results were consistent with experiments showing that the ECM symbiosis enhances the growth of host plants by promoting uptake of water and nutrients into hosts exposed to salt stress (Bandou et al. 2006; Bois et al. 2006a, b; Guerrero et al. 2019). Salt tolerance depends to a great extent on absorption and distribution of K and Na. The concentrations of Na clearly decreased and K content increased in ECM-plants compared to non-ECM plants, concomitantly with an increase of the K/Na ratio to maintain higher cell turgor (Bandou et al. 2006; Chen et al. 2017). Moreover, K ions are involved in regulating stomatal opening and osmotic potential in the vacuoles. The results obtained here have shown that the enhanced K uptake due to *S. bermudense* colonization can limit that of Na, thus improving the salt tolerance of ECM plants.

Alongside K, Ca is also an essential nutrient for plant growth (Evelin et al. 2019). Indeed, element analyses (Table S7) indicate that ECM plants had higher concentrations of Ca than non-ECM plants particularly under salt stress conditions. The Ca ion can also act as a second messenger in salt stress signaling (Munns and Tester 2008; Evelin et al. 2019). However, the absorption of Ca could be limited through a competition with an elevated Na concentration in the rhizosphere under salt stress (Evelin et al. 2019). Less Ca absorption and translocation than Na within the plant leads to a decrease in the Ca/Na ratio in salt stressed plants. Evelin et al. (2012) and Evelin et al. (2019) suggested that the Ca/Na ratio could also be increased by AM colonization and also suggested that Ca/Na ratio could be an indicator of salt tolerance in plants. This statement is consistent with Bullain Galardis et al. (2022), which showed a higher Ca/Na ratio in ECM than in non-ECM plants (Table S7). However, the mechanism involved is not well understood and needs further research (Evelin et al. 2019).

## Use of the ECM symbiosis between seagrape and *S. bermudense* in field conditions

The practice currently adopted in Cuba for ecological restoration purposes is based on planting local species for restoring the degraded coastal forest ecosystems. Seagrape is a good candidate because this native tree is salt, drought and wind-tolerant, stabilizing beaches and dunes (Parrotta 2000; Séne et al. 2015, 2018; Manokari et al. 2023). The ECM fungus *S. bermudense* can help seedlings seagrape to mitigate salt stress in nursery conditions (Bandou et al. 2006), whereas little is known about field performance of the inoculated plants. Nursery-grown seagrape pre-inoculated with spores of *S. bermudense* could contribute to the restoration of degraded coastal sand dunes in Cuba.

Planting of pre-inoculated seagrape seedlings was carried out in a degraded salty sandy coastal zone (0.3 ha) of the Manzanillo municipality in the Gulf of Guacanayabo (Bullain Galardis et al. 2023) where *C. uvifera* is absente. The high levels of salinity closer to the coastline did not impede the successful establishment and persistence of *S. bermudense* on roots of seagrape as demonstrated by the high level of ECM colonization (Bullain Galardis et al. 2023). One possible explanation of the fungal persistence is the ability of *S. bermudense* to tolerate salt stress as indicated in pure culture (Bâ et al. 2014) or in symbiosis with seagrape (Bandou et al. 2006; Bullaín Galardis et al. 2022; Bullain Galardis et al. 2023). Most of the studies used ECM inoculated plants at nursery stage prior to assessing their growth and survival in the field to measure restoration in terms of establishment and growth of inoculated compared to non-inoculated target plant hosts (Koske et al. 2008; Policelli et al. 2020). Another possible explanation is the lack of soil ECM inoculum potential in saline sites without seagrape, and leading to the subsequent absence of ECM colonization on uninoculated seagrape in the planting site. Indeed, ITS DNA barcoding revealed only bright white ECM fungal morphotype on inoculated seagrape in Manzanillo (Bullain Galardis et al. 2023) matched with *S. bermudense* sporocarps collected in other forest sites in Cuba where seagrape occurred (Bullain Galardis et al. 2024). However, pre-inoculated seagrape seedlings had been successfully colonized by *S. bermudense* in the planting site and required fungal inoculation prior to outplanting (Bullain Galardis et al. 2023).

Furthermore, pre-inoculation of seagrape significantly improved growth when compared to non-inoculated plants at each period of measurements (Table S8; Figure S6), a result congruent with ECM inoculation of conifers planted in oil sands subject to salt and drought stresses (Onwuchekwa et al. 2014). Zwiazek et al. (2019) showed a reduction of shoot Na content and high growth rates of ECM *P. contorta* growing in polluted urban environments by salt. Furthermore, survival was 100% in all seagrape treatments, suggestingECM fungal inoculation did not improve their field survival rates (Table S8). The beneficial effect of ECM inoculation in plant survival is rather mixed. Some studies reported no significant effect of ECM inoculation on survival rates of ECM plants after outplanting (Thomson et al. 1996; Selosse et al. 2000; Di Battista et al. 2002), whereas other reports showed significant, positive effects (Onwuchekwa et al. 2014). Different results in terms of survival rates of plants in field conditions could be due to differences in ECM fungal colonization of roots, competition with resident ECM fungi and/or ECM dependency of plants (Onwuchekwa et al. 2014).

Inoculation of *C. uvifera* seedlings by the ECM fungus *S. bermudense* also increased all physiological parameters measured every three months until 12 months and beyond after planting (Tables S9 and S10). The parameters photosynthetic rate (A), stomatal water conductance (gs), transpiration rate (E), sub-stomatal CO_2_ concentration (Ci), and water status (Ψwf and Ψwx) were significantly positively correlated with the improved growth of ECM seagrape exposed to salt stress (Table S9 and Table S10). This suggests that *S. bermudense* can elevate the A and E abilities, and water status of seagrape seedlings by increasing gs in field conditions. Our results are in agreement with the positive effect of *S. bermudense* on seagrape seedlings in nursery conditions (Bullain Galardis et al. 2022). Furthermore, our study is also consistent with work of Bai et al. (2021), which showed that high salt stress tended toward a lower significant decrease of A and E through a decrease in the gs, which was less important in ECM than non-ECM *Quercus mongolica*.

## Conclusions and future directions

In the Caribbean basin, seagrape ECM fungal diversity showed that (i) sporocarp survey weakly reflected the belowground ECM fungal community, although some fruiting species were also found on roots, (ii) the ECM fungal diversity was rather limited, but reminiscent of that from other ECM Polygonaceae, and (iii) seedlings and mature trees had overlapping ECM fungal communities, with *S. bermudense*, Thelephoraceae (*Tomentella* and *Thelephora*), and *Tuber* spp. dominating roots of both cohorts. Seedlings could be incorporated into a CMN maintained by understory mature trees where *S. bermudense* formed the main potential CMN between mature trees and seedlings. The role of the CMN is to be elucidated in regenerating seedlings of seagrape. However, the ECM fungus *S. bermudense* with seagrape appears to be a promising tool for restoring salinity-degraded coastal soils. The main physiological mechanisms for mitigating salt stress in seagrape were the reduction in Na concentration and the increase in K and Ca, with a higher K/Na and Ca/Na ratio, respectively, in the tissues of ECM seedlings compared with non-ECM seedlings. In addition, the beneficial effects of ECM symbiosis on photosynthesis and transpiration rates, fluorescence and chlorophyll content, stomatal conductance and water status improved the growth performance of seagrape provenances exposed to salt stress. From an ecological point of view, planting of the ECM seagrape not only may benefit the individual plant but, more importantly, may result in the development of ornamental plantings along roadsides and restoration programs of degraded coastal sand dunes in Cuba.

Further studies should be undertaken (i) to use homogeneous material from seagrape micropropagation, (ii) to assess the persistence of *S. bermudense*, as well as its potential impacts on soil and root-associated ECM fungal communities using high-throughput sequencing, (iii) to better understand how introduced *S. bermudense* interacts and coexists with the native microbiota community and whether this leads directly to changes in seagrape productivity, (iv) to determine if the ECM seagrape is potentially invasive out of their native area, (v) to retrace the history of seagrape using markers microsatellites to determine if the areas of origin and patterns of introduction of this plant match those of its fungal symbiont *S. bermudense*. All these questions remain challenges for promoting the use of ECM fungi in coastal areas where the sustainability of plant productivity is essential. We are currently investigating the use of ECM seagrape seedlings associated with *S. bermudense* in restoration programs where the plant and its ECM symbionts were absent, in order to increase the survival and establishment of this plant species in degraded coastal ecosystems in the Caribbean, Indian Ocean and West African regions.

## Electronic supplementary material

Below is the link to the electronic supplementary material.

Supplementary Material 1

## Data Availability

No datasets were generated or analysed during the current study.
