# Supplementary material for: Coccoloba uvifera L. associated with Scleroderma Bermudense Coker: a pantropical ectomycorrhizal symbiosis used in restoring of degraded coastal sand dunes
Source: Mycorrhiza. 2024 Oct 5;34(5-6):375–89. doi: 10.1007/s00572-024-01170-8 (PMC11604829; doi:10.1007/s00572-024-01170-8)
Supplement: Supplementary file 1 — Supplementary Material 1 [file 572_2024_1170_MOESM1_ESM.docx]

#### **Table S1**. Diversity of Coccoloba species in the Americas and the Caribbean islands (nd= not determined) (Bâ et al. 2014).

| Species | Synonymous | Distribution | Country | Habitat | Reference |
| --- | --- | --- | --- | --- | --- |
| *C. ascendens* | *Uvifera ascendens* | Fairly common | Guadeloupe, Martinique, South America, Trinidad, Dominica, St. Lucia, Grenade | Basaltic hills and forests | Fournet (2002) |
| *C. caravellae* | None | rare | Endemic to Martinique | nd | Fournet (2002) |
| *C. coronata* | *C. caribaea* | nd | Central America, Trinidad and Tobago, South America, Grenadines, Grenade | nd | Howard (1988) |
| *C. diversifolia* | *C. laurifolia* | nd | Florida, Mexico, Central America, Greater Antilles, Antigua | nd | Howard (1988) |
| *C. dussii* | None | Fairly common | Guadeloupe, Trinidad, St. Lucia, St. Vincent, Grenade | nd | Fournet (2002)  Howard (1988) |
| *C. krugii* | *Uvifera krugii* | rare | St. Martin, Bahamas, Greater Antilles, Anguilla, Guadeloupe, St. Christophe, St. Kitts | nd | Fournet (2002) |
| *C. pubescens* | *C. grandifolia, C. antiguensis, C. bonfilsiana* | Fairly common | Guadeloupe, Désirade, Marie-Galante, Martinique, Hispaniola, Porto Rico, Barbuda, Antigua, Nevis, Montserrat, Dominica, St. Lucia, Barbados | Coastal mesophytic,  Basaltic or calcareous hills | Fournet (2002)  Howard (1988) |
| *C. swartzii* | *C. diversifolia, C. barbadensis,*  *C. punctata,*  *Coccolobensis diversifolia, C. neglecta* | Common | Bahamas, Greater Antilles, Central America, South America, Antigua, Saba, St. Eustache, St. Christophe, St. Kitts, Nieves, Montserrat, Guadeloupe, Dominica, Marie-Galante, Martinique, St. Lucia, St. Vincent, Grenadines, Grenade, Barbados | Basaltic hills. Degraded xerophytic forest. | Fournet (2002)  Howard (1988) |
| *C. uvifera* | *Polygonum uvifera* | Very common | Bahamas, Greater Antilles, Mexico, Central America, South America, Anguilla, Antigua, Saba, St. Eustache, St. Bartelemy, St. Christophe, St. Kitts, Nieves, Montserrat, Guadeloupe, Dominica, Marie-Galante, Martinique, St. Lucia, St. Vincent, Grenadines, Grenade, Barbados | Sandy coast and sometimes rocky | Fournet (2002)  Howard (1988) |
| *C. venosa* | *C. nivea* | Quite rare | St. Martin, St. Barthelemy, Guadeloupe, Les Saintes, Marie-Galante, Martinique, Hispaniola, Porto Rico, Trinidad and Tobago, Venezuela, Antigua, Saba, St. Eustache, Montserrat, Dominica, St. Lucia, Grenadines, Grenade, Barbados | Coastal dry sand, tuff or basalt. Calcareous ills in *Pterocarpus* forest behind the mangrove swamp | Fournet (2002)  Howard (1988) |

#### **Table S2.** Sporocarp surveys associated with different Coccoloba species in the Greater and Lesser Antilles (Bâ et al. 2014).

| Fungal family | Fungal species | Host plant | Country | Reference |
| --- | --- | --- | --- | --- |
| Amanitaceae | *Amanita cystidiosa* | *C. uvifera* | Anguilla, Guana (Virgin Islands), Puerto Rico | Miller et al. 2000 |
|  | *A. antillana* | *C. diversifolia, C. pubescens* | Martinique, Trinidad | Pegler 1983 |
|  | *A. arenicola* | *C. uvifera* | Puerto Rico, Guadeloupe | Miller et al. 2000, Sène et al. 2015 |
|  | *A. microspora* | *C. uvifera* | Puerto Rico | Miller et al. 2000 |
|  | *A. craseoderma*  *Amanita* sp. | *C. pubescens*  *C. uvifera* | Martinique  Cuba | Pegler 1983  Bullain Galardis et al. 2024 |
| Boletaceae | *Austrogautieria* sp. | *C. pubescens, C. swartzii* | Martinique | Bâ et al. 2014 |
|  | *Boletus roborculus* | *Coccoloba* sp. | Porto Rico | Miller et al. 2000 |
|  | *Boletelus cubensis* | *C. pubescens, C. swartzii* | Martinique | Bâ et al. 2014 |
|  | *Fistulinella gloeocarpa* | *C. diversifolia* | Martinique | Pegler 1983, Bâ et al. 2014 |
|  | *Limacella* sp. | *C. pubescens, C. swartzii* | Martinique | Pegler 1983, Bâ et al. 2014 |
|  | *Melanogaster* spp. | *C. pubescens, C. swartzii* | Martinique, Guadeloupe | Bâ et al. 2014, Sène et al. 2015 |
|  | *Xerocomus hypoxanthus* | *C. uvifera* | Martinique | Pegler 1983 |
|  | *X. coccolobae* | *C. uvifera, C. pubescens, C. swartzii* | Martinique | Pegler 1983 |
|  | *X. guadelupae* | *Coccoloba* sp. | Martinique, Guadeloupe, Dominica | Pegler 1983 |
|  | *X. cuneipes* | *C. uvifera* | Martinique | Pegler 1983 |
|  | *Scleroderma bermudense* |  | Puerto Rico, Guadeloupe, Martinique, Cuba | Guzman et al. 2004, Sène et al. 2015  Bullain Galardis et al. 2024 |
|  | *S. stellatum* | *Coccoloba* sp.,  *C. uvifera* | Cuba, Puerto Rico | Kreisel 1971 |
|  | *Scleroderma* sp*.* | *C. swartzii, C. pubescens* | Martinique | Bâ et al. 2014 |
| Cantharellaceae | *Cantharellus cinnabarinus*  *Cantharellus* sp. | *C. uvifera*  *C. uvifera* | Martinique, Guadeloupe, Puerto Rico, Cuba  Cuba | Pegler 1983, Sène et al. 2015  Bullain Galardis et al. 2024 |
| Cortinariaceae | *Inocybe littoralis* | *C. uvifera* | Martinique, Guadeloupe | Pegler 1983, Sène et al. 2015 |
|  | *I. xerophytica* | *C. uvifera* | Guadeloupe | Pegler 1983, Sène et al. 2015 |
| Gomphaceae | *Ramaria cyanocephala* | *C. swartzii, C. pubescens* | Martinique | Bâ et al. 2014 |
| Hymenochaetaceae | *Coltricia* sp. | *C. swartzii, C. pubescens* | Martinique | Bâ et al. 2014 |
| Inocybaceae | *Inocybe littoralis* | *C. uvifera* | Martinique, Guadeloupe | Pegler 1983, Bâ et al. 2014, Sène et al. 2015 |
|  | *I. xerophytica* | *C. uvifera* | Martinique, Guadeloupe | Pegler 1983, Bâ et al. 2014, Sène et al. 2015 |
| Russulaceae | *Inocybe* sp.  *Lactarius coccolobae* | *C. uvifera*  *C. uvifera* | Cuba  Anguilla, Guana (Virgin Islands), Puerto Rico | Bullain Galardis et al. 2024  Miller et al. 2000 |
|  | *L. caribaeus* | *C. pubescens, C. diversifolia* | Martinique | Pegler 1983 |
|  | *L. castaneibadius* | *C. diversifolia, C. swartzii,*  *C. pubescens* | Martinique | Pegler 1983, Bâ et al. 2014 |
|  | *L. hygrophoroides* | *C. pubescens* | Martinique | Pegler 1983 |
|  | *L. murinipes* | *Coccoloba* sp. | Martinique | Pegler 1983 |
|  | *L. nebulosus* | *C. pubescens, C. diversifolia* | Martinique | Pegler 1983 |
|  | *Russula cremeolilacina* | *C. diversifolia, C. uvifera* | Martinique, Guadeloupe | Pegler 1983, Sène et al. 2015 |
|  | *R. brevipes* | *C. swartzii* | Martinique | Pegler 1983 |
|  | *R. littoralis* | *C. uvifera* | Martinique, Virgin Islands, Puerto Rico |  |
|  | *R. martinica* | *C. diversifolia* | Martinique | Pegler 1983 |
|  | *R. metachromaticae*  *Russula* sp. | *C. uvifera*  *C. uvifera* | Martinique  Cuba | Pegler 1983  Bullain Galardis et al. 2024 |
| Thelephoraceae | *Thelephora* sp. | *C. swartzii, C. pubescens* | Martinique | Bâ et al. 2014 |

**Figure S5.** Tolerance of *Scleroderma bermudense*, *Melanogaster* sp. and *Scleroderma* sp. to salt stress *in vitro* conditions,

(a) radial growth, (b) biomass yield, (c) Na contents, (d) proline contents. Two-way analysis (salinity and

inoculation) of variance was performed. For each treatment, bars topped with different letters are significant

different according to the Tukey HSD test at P ≤ 0.05. Vertical bars indicate standard errors of mean value (n = 10).

(Bâ et al. 2014)

a

c

d

b

**Table S3.** Effect of provenance, inoculation and salinity on some morphological variables in seagrape seedlings in nursery conditions (Bullain Galardis et al. 2022).

Three-way analysis (provenance, inoculation and salinity) of variance was performed. For each treatment, different letters are significant

different according Tukey’s test at P ≤ 0.05 with the InfoStat software version 2008. Values are the means ±SD (n =10).

| Provenance of *C. uvifera* | Inoculation treatment | Salinity  (dS m^-1^) | Stem length  (cm) | Stem  diameter  (mm) | Number of leaves | Root length  (cm) | Total biomass (g) | Leaf area  (cm^2^) | ECM  dependency  (%) |
| --- | --- | --- | --- | --- | --- | --- | --- | --- | --- |
| Las Coloradas | Non-mycorrhizal | 0.02 | 16.1±1.3fg | 3.3±0.2cd | 5.6±0.5efg | 26.8±1.8ab | 2.7±0.2cd | 100.4±31.5bc | - |
|  |  | 5 | 13.7±0.8cdef | 3.0±0.5bc | 4.2±0.4cd | 27.9±2.7abc | 2.4±0.4bc | 72.4±21.4c | - |
|  |  | 15 | 11.0±1.0abc | 2.3±0.2a | 3.4±0.5bc | 30.4±2.8bcd | 1.2±0.3a | 41.8±6.0d | - |
|  |  | 25 | 9.0±0.4a | 2.2±0.2a | 2.40±0.5ab | 27.0±1.4ab | 0.9±0.3a | 41.9±8.1d | - |
|  | Mycorrhizal | 0.02 | 19.4±2.1h | 4.9±0.2g | 7.4±0.5i | 41.0±3.6e | 4.0±0.6e | 237.7±43.1a | 30.6±8.9abc |
|  |  | 5 | 18.2±2.0gh | 4.1±0.1ef | 6.8±0.4hi | 40.0±2.4e | 3.0±0.3d | 142.6±22.9ab | 19.3±21.9a |
|  |  | 15 | 14.2±1.4def | 3.6±0.22de | 5.8±0.4fgh | 37.2±4.5de | 2.2±0.2b | 97.2±3.2abc | 43.9±14.8bc |
|  |  | 25 | 12.6±1.1bcde | 3.0±0.1c | 5.2±0.45def | 37.3±3.5de | 1.9±0.37b | 81.3±17.3bc | 47.7±20.4bc |
| Punta de Tomate | Non mycorrhizal | 0.02 | 15.1±1.6ef | 3.9±0.1cd | 5.4±0.5ef | 30.5±1.4bcd | 2.9±0.16cd | 99.9±32.0bc | - |
|  |  | 5 | 12.9±0.5bcde | 3.3±0.2cd | 5.0±0.0def | 31.0±2.1bcd | 2.4±0.3bc | 71.7±21.4c | - |
|  |  | 15 | 10.9±0.1ab | 2.4±0.2ab | 3.2±0.4abc | 28.1±3.6abc | 1.3±0.2a | 40.9±5.1d | - |
|  |  | 25 | 8.9±0.3a | 1.92±0.2a | 2.2±0.4a | 20.8±3.2a | 0.9±0.1a | 42.0±7.9d | - |
|  | Mycorrhizal | 0.02 | 18.9±1.4h | 4.6±0.2fg | 7.0±0.0i | 35.6±4.5cde | 3.9±0.3e | 239.0±42.4a | 29.8±7.2abc |
|  |  | 5 | 17.9±1.4gh | 4.0±0.5ef | 6.6±0.5ghi | 36.9±4.4de | 3.0±0.3d | 141.4±20.2ab | 19.8±10.6ab |
|  |  | 15 | 13.9±0.9def | 2.9±0.1cd | 5.6±0.5efg | 34.5±6.4bcde | 2.4±0.2bc | 96.8±2.9abc | 46.9±10.9bc |
|  |  | 25 | 11.9±0.5bcd | 2.9±0.2bc | 4.6±0.5de | 33.8±2.4bcde | 1.9±0.3b | 80.5±17.1bc | 48.8±8.9c |
| Provenance | | | NS | NS | NS | S | NS | NS | NS |
| Inoculation | | | S | S | S | S | S | S | S |
| Salinity | | | S | S | S | S | S | S | S |
| Provenance x Inoculation | | | NS | S | NS | S | NS | NS | NS |
| Provenance x Salinity | | | NS | NS | NS | NS | NS | NS | NS |
| Inoculation x Salinity | | | NS | S | S | NS | S | S | S |
| Provenance x Inoculation x Salinity | | | NS | NS | NS | S | NS | NS | NS |

.

#### **Table S4.** Effect of provenance, inoculation and salinity on stomatal conductance (gs), photosynthetic (A) and transpiration (E) rates and sub-stomatal CO_2_ (Ci)

in leaves of seagrape seedlings in nursery conditions (Bullain Galardis et al. 2022). Three-way analysis (provenance, inoculation and salinity) of variance was performed.

For each treatment, different letters are significant different according Tukey’s test at P ≤ 0.05 with the InfoStat software version 2008. Values are the means ±SD (n =10).

| Provenance of *C. uvifera* | Inoculation treatment | Salinity  (dS m^-1^) | gs  (mol m^-2^ s^-1^) | E  (mol m^-2^ s^-1^) | A  (µmol m^-2^ s^-1^) | Ci  (vpm) | |
| --- | --- | --- | --- | --- | --- | --- | --- |
| Las Coloradas | Non- mycorrhizal | 0.02 | 0.1±0.0cd | 0.96±0.22cd | 1.26±0.09d | 374.80±1.23e |  |
|  |  | 5 | 0.1±0.0bc | 0.54±0.14ab | 0.86±0.07c | 345.80±1.03c |  |
|  |  | 15 | 0.0±0.0ab | 0.49±0.02ab | 0.50±0.03b | 325.10±0.88b |  |
|  |  | 25 | 0.0±0.1ab | 0.43±0.04a | 0.22±0.02a | 274.50±1.08a |  |
|  | Mycorrhizal | 0.02 | 0.2±0.0f | 2.61±0.25e | 1.96±0.02e | 432.10±1.85g |  |
|  |  | 5 | 0.1±0.1ef | 1.93±0.06de | 1.78±0.13e | 431.00±1.25g |  |
|  |  | 15 | 0.1±0.0cde | 1.07±0.09cd | 0.89±0.05c | 394.80±1.23f |  |
|  |  | 25 | 0.1±0.0c | 0.71±0.05bc | 0.57±0.01b | 361.90±1.60d |  |
| Punta de Tomate | Non- mycorrhizal | 0.02 | 0.1±0.0cd | 0.95±0.26cd | 1.25±0.11d | 376.10±1.20e |  |
|  |  | 5 | 0.0±0.0ab | 0.52±0.16ab | 0.83±0.08c | 346.40±0.84c |  |
|  |  | 15 | 0.0±0.01a | 0.47±0.07a | 0.51±0.05b | 323.80±0.79b |  |
|  |  | 25 | 0.0±0.01a | 0.41±0.06a | 0.19±0.05a | 273.90±0.88a |  |
|  | Mycorrhizal | 0.02 | 0.2±0.0f | 2.60±0.27e | 1.91±0.02e | 431.70±1.57g |  |
|  |  | 5 | 0.1±0.0def | 1.91±0.08de | 1.79±0.15e | 430.80±1.03g |  |
|  |  | 15 | 0.1±0.0bc | 1.05±0.09cd | 0.87±0.09c | 396.10±0.74f |  |
|  |  | 25 | 0.1±0.0cd | 0.69±0.08bc | 0.54±0.05b | 363.10±0.88d |  |
| Provenance | | | S | NS | NS | NS |  |
| Mycorrhization | | | S | S | S | S |  |
| Salinity | | | S | S | S | S |  |
| Provenance x Mycorrhization | | | NS | NS | NS | NS |  |
| Provenance x Salinity | | | NS | NS | NS | NS |  |
| Mycorrhization x Salinity | | | S | S | S | S |  |
| Provenance x Mycorrhization x Salinity | | | NS | NS | NS | S |  |

#### **Table S5.** Effect of provenance, inoculation and salinity on water status in leaves of seagrape seedlings

in nursery conditions (Bullain Galardis et al. 2022). Three-way analysis (provenance, inoculation and salinity) of

variance was performed. For each treatment, different letters are significant different according

Tukey’s test at P ≤ 0.05 with the InfoStat software version 2008. Values are the means ±SD (n =10).

| Provenance of *C. uvifera* | Inoculation treatment | Salinity  (dS m^-1^) | RWC  (%) | Ψwf  (MPa) | Ψwx  (MPa) |
| --- | --- | --- | --- | --- | --- |
| Las Coloradas | Non-mycorrhizal | 0.02 | 77.1±1.4efgh | -0.7±0.0b | -0.5±0.0b |
|  |  | 5 | 88.6±4.0ij | -1.5±0.0d | -1.0±0.0d |
|  |  | 15 | 68.9±2.9cde | -2.5±0.0f | -1.9±0.1f |
|  |  | 25 | 58.2±7.2ab | -4.0±0.0h | -3.1±0.1h |
|  | Mycorrhizal | 0.02 | 93.7±3.7jk | -0.5±0.0a | -0.3±0.0a |
|  |  | 5 | 98.5±5.3k | -0.9±0.0c | -0.7±0.4c |
|  |  | 15 | 78.8±4.3fgh | -2.0±0.0e | -1.4±0.0e |
|  |  | 25 | 74.6±2.1defg | -3.3±0.0g | -2.4±0.1g |
| Punta de Tomate | Non-mycorrhizal | 0.02 | 69.1±2.9cde | -0.7±0.0b | -0.5±0.0b |
|  |  | 5 | 80.6±2.5ghi | -1.5±0.0d | -1.1±0.0d |
|  |  | 15 | 60.9±4.4bc | -2.5±0.0f | -1.9±0.1f |
|  |  | 25 | 50.2±5.7a | -4.0±0.0h | -3.2±0.0h |
|  | Mycorrhizal | 0.02 | 85.7±5.1hij | -0.5±0.0a | -0.3±0.0a |
|  |  | 5 | 90.5±3.8jk | -1.0±0.1c | -0.7±0.1c |
|  |  | 15 | 70.8±5.9def | -2.0±0.1e | -1.5±0.0e |
|  |  | 25 | 66.8±0.7bcd | -3.4±0.1g | -2.5±0.0g |
| Provenance | | | S | S | S |
| Inoculation | | | S | S | S |
| Salinity | | | S | S | S |
| Provenance x Inoculation | | | NS | NS | NS |
| Provenance x Salinity | | | NS | NS | NS |
| Inoculation x Salinity | | | S | S | S |
| Provenance x Inoculation x Salinity | | | NS | NS | NS |

#### **Table S6.** Effect of provenance, inoculation and salinity on the K/Na ratio in leaves, stems and roots of seagrape seedlings

in nursery conditions (Bullain Galardis et al. 2022). Three-way analysis (provenance, inoculation and salinity) of

variance was performed. For each treatment, different letters are significant different according

Tukey’s test at P ≤ 0.05 with the InfoStat software version 2008. Values are the means ±SD (n =10).

| Provenance of *C. uvifera* | Inoculation treatment | Salinity  (dS m^-1^) | Ratio K/Na (mg/l) | | |
| --- | --- | --- | --- | --- | --- |
|  |  |  | Leaves | Stems | Roots |
| Las Coloradas | Non-mycorrhizal | 0.02 | 0.8±0.0d | 1.1±0.0d | 0.7±0.0d |
|  |  | 5 | 0.8±0.0c | 0.9±0.0c | 0.7±0.0c |
|  |  | 15 | 0.4±0.0b | 0.4±0.0b | 0.6±0.0b |
|  |  | 25 | 0.2±0.0a | 0.3±0.0a | 0.3±0.0a |
|  | Mycorrhizal | 0.02 | 2.6±0.0i | 6.3±0.0h | 1.7±0.0h |
|  |  | 5 | 2.4±0.0gh | 2.3±0.0g | 1.2±0.0g |
|  |  | 15 | 1.9±0.0f | 2.0±0.0f | 0.9±0.0f |
|  |  | 25 | 1.2±0.0e | 1.6±0.0e | 0.8±0.0e |
| Punta de Tomate | Non-mycorrhizal | 0.02 | 0.8±0.0d | 1.2±0.1d | 0.7±0.0d |
|  |  | 5 | 0.8±0.0c | 0.9±0.1c | 0.7±0.0c |
|  |  | 15 | 0.4±0.0b | 0.4±0.0b | 0.7±0.0b |
|  |  | 25 | 0.2±0.0a | 0.3±0.0a | 0.3±0.0a |
|  | Mycorrhizal | 0.02 | 2.5±0.2hi | 6.5±0.1h | 1.7±0.0h |
|  |  | 5 | 2.3±0.1g | 2.4±0.0g | 1.2±0.0g |
|  |  | 15 | 1.9±0.0f | 2.1±0.0f | 0.9±0.0f |
|  |  | 25 | 1.3±0.0e | 1.1±0.1e | 0.8±0.1e |
| Provenance | | | NS | NS | NS |
| Inoculation | | | S | S | S |
| Salinity | | | S | S | S |
| Provenance x Inoculation | | | NS | NS | NS |
| Provenance x Salinity | | | S | S | NS |
| Inoculation x Salinity | | | S | S | S |
| Provenance Inoculation x Salinity | | | S | S | S |

#### **Table S7.** Effect of provenance, inoculation and salinity on the Ca/Na ratio in leaves, stems and roots of seagrape

seedlings in nursery conditions (Bullain Galardis et al. 2022). Three-way analysis (provenance, inoculation and salinity) of

variance was performed. For each treatment, different letters are significant different according

Tukey’s test at P ≤ 0.05 with the InfoStat software version 2008. Values are the means ±SD (n =10).

| Provenance of *C. uvifera* | Inoculation treatment | Salinity  (dS m^-1^) | Ratio Ca/Na (mg/l) | | |
| --- | --- | --- | --- | --- | --- |
|  |  |  | Leaves | Stems | Roots |
| Las Coloradas | Non-mycorrhizal | 0.02 | 2.1±0.1d | 5.9±0.1d | 4.2±0.1d |
|  |  | 5 | 1.9±0.1c | 4.9±0.0c | 3.6±0.0c |
|  |  | 15 | 1.0±0.0b | 2.2±0.0b | 2.6±0.0b |
|  |  | 25 | 0.4±0.0a | 1.9±0.0a | 1.5±0.1a |
|  | Mycorrhizal | 0.02 | 6.2±0.0h | 16.6±0.2h | 11.2±0.2h |
|  |  | 5 | 5.2±0.0g | 12.7±0.1g | 7.0±0.1g |
|  |  | 15 | 4.5±0.1f | 10.1±0.0f | 5.5±0.1f |
|  |  | 25 | 3.2±0.0e | 7.8±0.0e | 4.4±0.1e |
| Punta de Tomate | Non-mycorrhizal | 0.02 | 2.1±0.0d | 6.3±0.4d | 4.3±0.1de |
|  |  | 5 | 1.8±0.0c | 4.6±0.1c | 3.7±0.0c |
|  |  | 15 | 1.0±0.0b | 2.4±0.0b | 2.6±0.0b |
|  |  | 25 | 0.4±0.0a | 1.8±0.0a | 1.5±0.0a |
|  | Mycorrhizal | 0.02 | 5.9±0.5h | 17.0±0.4h | 11.2±0.0h |
|  |  | 5 | 5.1±0.2g | 12.9±0.1g | 6.9±0.0g |
|  |  | 15 | 4.4±0.1f | 12.9±0.1f | 5.5±0.0f |
|  |  | 25 | 3.4±0.1e | 12.9±0.1e | 4.3±0.0de |
| Provenance | | | NS | NS | NS |
| Inoculation | | | S | S | S |
| Salinity | | | S | S | S |
| Provenance x Inoculation | | | NS | NS | NS |
| Provenance x Salinity | | | S | S | NS |
| Inoculation x Salinity | | | S | S | S |
| Provenance x Inoculation x Salinity | | | NS | S | NS |

#### **Table S8.** Effect of inoculation and month after planting on some morphological variables in seagrape seedlings

in field conditions (Bullain Galardis et al. 2023). Two-way analysis (Month of planting and inoculation) of

variance was performed. For each treatment, different letters are significant different according

Tukey’s test at P ≤ 0.05 with the InfoStat software version 2008. Values are the means ±SD (n =25).

| Month after  Planting | Inoculation  Treatment | Survival  rate  (%) | Number of  Leaves | Collar  diameter  (mm) | Stem  length  (cm) |
| --- | --- | --- | --- | --- | --- |
| 3 | Non-inoculated | 100 | 3.4±0.7g | 2.6±0.4g | 10.8±1.1f |
|  | Inoculated | 100 | 5.9±0.8f | 3.7±0.3f | 13.9±2.6e |
| 6 | Non-inoculated | 100 | 7.5±0.6ef | 4.8±0.2e | 16.7±1.3e |
|  | Inoculated | 100 | 7.9±0.6de | 6.3±0.1d | 19.9±2.6d |
| 9 | Non-inoculated | 100 | 9.4±0.6cd | 6.4±0.1d | 22.3±0.8d |
|  | Inoculated | 100 | 11.0±0.5c | 8.3±0.1c | 27.3±0.6c |
| 12 | Non-inoculated | 100 | 23.4±3.9b | 26.6±0.8b | 58.0±6.8b |
|  | Inoculated | 100 | 49.2±5.1a | 39.3±0.7a | 115.3±5.6a |
| Inoculation |  |  | P= 0.0001 | P= 0.0001 | P= 0.0001 |
| Month |  |  | P= 0.0001 | P= 0.0001 | P= 0.0001 |
| Inoculation x Month | |  | P= 0.0001 | P= 0.0001 | P= 0.0001 |

#### **Table S9.** Effect of inoculation and month after planting on photosynthetic (A) and transpiration (E) rates, stomatal

#### conductance (gs), and sub-stomatal CO_2_ (Ci) in leaves of seagrape seedlings in field conditions (Bullain Galardis et al. 2023).

Two-way analysis (Month of planting and inoculation) of variance was performed. For each treatment, different

letters are significant different according Tukey’s test at P ≤ 0.05 with the InfoStat software version 2008.

Values are the means ±SD (n =25).

| Month after  planting | Inoculation  treatment | A  (µmol m^-2^ s^-1^) | E  (mol m^-2^ s^-1^) | gs  (mol m^-2^ s^-1^) | Ci  (vpm) |
| --- | --- | --- | --- | --- | --- |
| 3 | Non-inoculated | 0.6±0.1h | 0.5±0.0h | 0.0±0.0g | 326.2±4.9f |
|  | Inoculated | 1.0±0.1g | 1.4±0.3g | 0.1±0.0f | 414.3±6.9c |
| 6 | Non-inoculated | 2.5±0.5f | 2.9±0.5f | 0.1±0.0f | 348.1±5.1e |
|  | Inoculated | 3.5±0.5e | 3.8±0.6e | 0.2±0.0d | 418.8±4.2b |
| 9 | Non-inoculated | 5.6±0.7d | 5.9±0.3d | 0.2±0.0d | 365.4±5.1d |
|  | Inoculated | 6.7±0.7c | 6.9±0.2c | 0.3±0.0b | 420.0±4.0b |
| 12 | Non-inoculated | 9.6±1.5b | 8.7±0.5b | 0.2±0.0d | 368.3±5.5d |
|  | Inoculated | 10.9±1.4a | 9.7±0.5a | 0.3±0.0b | 424.4±4.4a |
| Inoculation |  | P= 0.0001 | P= 0.0001 | P= 0.0001 | P= 0.0001 |
| Month |  | P= 0.0001 | P= 0.0001 | P= 0.0001 | P= 0.0001 |
| Inoculation x Month | | P= 0.061 | P= 0.461 | P= 0.346 | P= 0.0001 |

#### **Table S10**. Effect of inoculation and month after planting on foliar (Ψwf) and xylem (Ψwx) water potential in leaves

of seagrape seedlings in field conditions (Bullain Galardis et al. 2023). Two-way analysis (Month of planting and inoculation)

of variance was performed. For each treatment, different letters are significant different according Tukey’s test at P ≤ 0.05

with the InfoStat software version 2008. Values are the means ±SD (n =25).

| Month after  planting | Inoculation  treatment | Ψwf  (MPa) | Ψwx  (MPa) |
| --- | --- | --- | --- |
| 3 | Non-inoculated | -2.4±0.2e | -1.9±0.6e |
|  | Inoculated | -1.7±0.5b | -1.2±0.4bc |
| 6 | Non-inoculated | -2.3±0.2de | -1.6±0.5de |
|  | Inoculated | -1.2±0.1a | -0.9±0.3ab |
| 9 | Non-inoculated | -2.1±0.1cd | -1.4±0.6cd |
|  | Inoculated | -1.1±0.1a | -0.9±0.4ab |
| 12 | Non-inoculated | -2.0±0.1c | -1.1±0.2bc |
|  | Inoculated | -1.1±0.1a | -0.6±0.1a |
| Inoculation |  | P=0.0001 | P=0.0001 |
| Month |  | P=0.0001 | P=0.0001 |
| Inoculation x Month | | P=0.0001 | P=0.458 |

**Figure S6.** A partial view of the inoculated (a) *vs* non-inoculated (b) seagrape at 12 months in field conditions at Cuba

(Bullain Galardis et al. 2023).


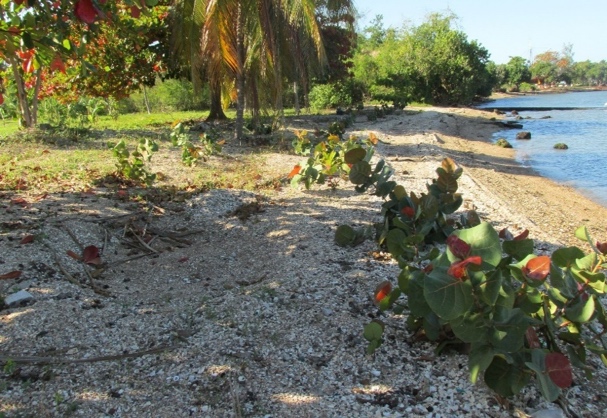


**(a)**

**(b)**
